# Dilatation and Curettage after Uterine Artery Embolization versus Methotrexate Injection for the Treatment of Cesarean Scar Pregnancy: A Single-Center Experience

**DOI:** 10.3390/medicina60030487

**Published:** 2024-03-15

**Authors:** Basilio Pecorino, Giuseppe Scibilia, Bianca Mignosa, Maria Cristina Teodoro, Benito Chiofalo, Paolo Scollo

**Affiliations:** 1 Obstetrics and Gynecology Division, Umberto I Hospital, Kore University of Enna, 94100 Enna, Italy; basilio.pecorino@unikore.it; 2 Obstetrics and Gynecology Division, Giovanni Paolo II Hospital, 97100 Ragusa, Italy; g.scibilia@libero.it; 3 Maternal and Child Department, Obstetrics and Ginecology Division, Cannizzaro Hospital, 95126 Catania, Italy; bianca.mignosa@gmail.com (B.M.); cris.teo85@gmail.com (M.C.T.); 4 Maternal and Child Department, Obstetrics and Ginecology Division, Cannizzaro Hospital Catania, Kore University of Enna, 94100 Enna, Italy; paolo.scollo@unikore.it

**Keywords:** cesarean scar pregnancy, ectopic pregnancy, methotrexate, uterine artery embolization, uterine curettage

## Abstract

*Background and Objectives*: Cesarean scar pregnancy (CSP) represents a type of ectopic pregnancy in which the embryo implants inside the scar of a previous cesarean section. This condition can lead to maternal morbidity and mortality. The best therapeutic approach in terms of clinical effectiveness and patient safety for CSP has not been described yet, although different therapeutic strategies are currently available. The purpose of the present study was to analyze the success rate of two different treatments in a single institution. *Materials and Methods*: A retrospective study was performed among patients diagnosed with CSP at the Gynecology and Obstetrics Department of the “Cannizzaro” Hospital in Catania (University of Enna—Italy) from January 2016 to December 2022. The diagnosis was made by 2D/3D transvaginal ultrasound, following Timor-Tritsch criteria. Two treatment strategies were performed: local and systemic methotrexate (MTX) injection and uterine artery embolization (UAE) with subsequent dilatation and curettage (D&C). All treated women underwent subsequent clinical and sonographic follow-up. Complete recovery was defined as the reduction of β-HCG values until it was undetectable and the disappearance of the mass in the uterine scar on ultrasound. *Results*: Nineteen patients were included; nine were in the MTX group and ten were in the UAE + D&C group. No significant differences were found between the two groups in terms of clinical parameters. Treatment was successful in 4 of 10 (44%) patients in the MTX group and 10 of 10 (100%) in the UAE + D&C group (*p* = 0.01); the length of hospital stay was significantly shorter in the latter group (*p* < 0.0001). *Conclusions*: In our experience, administration of MTX is not recommended as the primary treatment or pre-treatment. Dilatation and curettage after uterine artery embolization are better than methotrexate injection for the treatment of cesarean scar pregnancy in a single-institution series in terms of complete recovery and length of hospital stay.

## 1. Introduction

An ectopic pregnancy occurs when the blastocyst implants outside the uterine cavity, typically in the fallopian tube or more unusual instances, such as the abdominal cavity, the ovarian surface, the cervical canal, and the scar of a previous cesarean section or a previous myomectomy [1,2].

Different types of ectopic pregnancies have been described, depending on the localization of the implantation. Risk factors for ectopic pregnancies are not completely known; previous endometrial and pelvic infections have a role in the pathogenesis of this serious medical condition [1].

Cesarean scar pregnancy (CSP) is an uncommon type of ectopic pregnancy in which the gestational sac implants inside the scar of a previous uterine section [3]. The most important risk factor for CSP is cesarean section; previous myomectomy has less impact on the pathogenesis of CSP. Although CSP is considered a rare type of ectopic pregnancy, its incidence is gradually increasing; it has almost doubled since 2000, due to the rising rate of cesarean sections worldwide [4]. CSP can rarely progress until a successful live birth, but it causes a life-threatening risk for women, as it can lead to heightened maternal morbidity and mortality due to complications such as severe bleeding, uterine rupture, and retained products of conception [5]. The CSP is typically located in the scar defect located at the lower anterior uterine wall. An early diagnosis, made possible through transvaginal ultrasound and pelvic magnetic resonance imaging (MRI), is crucial for enhancing CSP management, preventing serious complications, and preserving the woman’s future reproductive potential [6,7].

Several classifications have been proposed, basically based on the direction of the growth of CSP; usually, type 1 and type 2 CSP are recognized [2,3,4]. In type 1, also known as endogenic CSP, the pregnancy is implanted on the scar and grows within the uterine cavity; in type 2 or exogenic, the blastocyst implants into the scar and grows toward the bladder or abdominal cavity, which may lead to more serious complications than in type 1 [2,3,4]. Despite recent significant progress in diagnostic and surgical strategies for CSP, the fundamental treatment for this rare medical condition continues to be the termination of pregnancy [5].

The best therapeutic approach in terms of clinical effectiveness and patient safety for CSP has not been described yet, although different therapeutic strategies are currently available [8]. Treatment options include conservative medical approaches such as systemic and/or local methotrexate (MTX) injection, local injection of sclerosant drugs like lauromacrogol, or surgical procedures like hysteroscopy, laparoscopic surgery, dilatation and curettage (D&C), and uterine artery embolization (UAE), as well as demolition treatments like hysterectomy [9,10,11]. These therapies can be used individually or in combination [12].

Since there are currently no treatment guidelines for managing CSP, the aim of this study is to analyze the clinical outcomes resulting from CSP treatments performed by our team during different years. Specifically, we aim to compare the results between a conservative medical treatment (using MTX) and a conservative surgical approach, using a combination of uterine artery embolization associated with uterine dilatation and curettage, to assess the more effective therapeutic option and identify criteria for making the best treatment choice for patients affected by this serious medical condition.

## 2. Materials and Methods

### 2.1. Patients

This is a retrospective study carried out at the Gynecology and Obstetrics University Department of the Cannizzaro Hospital of Catania (Kore University of Enna—Italy). The period of study is from January 2016 to December 2022, as we started to perform UAE + D&C in our hospital since 2016. Clinical and anamnestic data were collected from patients’ medical records.

Patients included in the study met the following selection criteria: age > 18 years old, diagnosis of CSP made by transvaginal ultrasound, clinical history of cesarean section, clinical manifestations, and treatment with MTX or UAE + D&C. Exclusion criteria were minor age, other treatments than those under study, and CSP arising after myomectomy.

Sonographic examinations were performed with a GE VOLUSON E8 (General Electric Co., Boston, MA, USA). The diagnosis of CSP met the following criteria: no gestational sac appears in the uterus and the cervical canal; a gestational sac or mass located in the anterior wall of the isthmic portion; a gestational sac embedded within the myometrium, with an absence or defect in the myometrium between the bladder and the sac; evidence of functional trophoblastic/placental circulation on Doppler scans; and a negative sliding organ sign, defined as the inability to displace the gestational sac from its position at the level of the internal os [10,11,12,13].

In our hospital, ultrasound examinations were conducted by several specific experienced gynecologists to ensure precise description, including the largest dimensions of the gestational sac in terms of its length, width, and height; the thinnest myometrial layer; the vascular pattern in the scar (rich or not rich); the CSP type (type 1: in the niche, type 2: on top of the scar); and the presence or absence of fetal cardiac activity.

### 2.2. Treatments

Two treatment strategies were compared in this study: local and systemic MTX injection and uterine artery embolization (UAE) with subsequent dilatation and curettage (D&C). As we do not have guidelines or even reliable scientific evidence for the treatment of CSP, the choice of treatment has depended on the availability of interventional radiology and the patient’s and operators’ preferences.

All patients in the MTX group were treated with MTX injections. The MTX injection was carried out either locally, via injection of 50 mg of MTX or 2 cc MTX + KCl in a 2:1 ratio directly into the ovular cavity, under ultrasound guidance, or systemically, via an intramuscular injection of 100 mg of MTX. On the other hand, patients in the UAE + D&C group were treated as follows: after local anesthesia, a 4F angiographic catheter was inserted into the right femoral artery and extended into the uterine arteries bilaterally. Fifty-to-one hundred milligrams of an embolic agent with gelatin sponge particles for unambiguous embolization were injected after confirmation of the catheters’ correct position. After UAE, D&C was performed within 24 h of UAE. Dilatation of the uterine cervix was carried out with Hegar dilators; after that, hysterosuction was used to remove most of the chorionic residues; and finally, an ultrasound-guided complete curettage of the uterine cavity was performed.

All treated women underwent subsequent clinical and sonographic follow-up. Cases with complete recovery and without adverse events or the need for adjuvant treatments were considered successful. The main adverse events were massive bleeding and pelvic pain, while the adjuvant treatments analyzed included the use of further doses of drugs, such as MTX, or the use of surgery. Complete recovery was defined as the reduction of β-HCG values until it was undetectable and the disappearance of the mass in the uterus on ultrasound.

The study was approved by the Ethical Committee of Cannizzaro Hospital (IRB06/22). The design, analysis, interpretation of data, drafting, and revisions are in compliance with the Helsinki Declaration, the Committee on Publication Ethics guidelines, and the Strengthening the Reporting of Observational Studies in Epidemiology (STROBE) Statement [14], validated by the Enhancing the Quality and Transparency of Health Research (EQUATOR) Network. The study was not advertised, and no remuneration was offered to encourage patients to give consent for the collection and analysis of their data. Each patient enrolled in this study was informed about the aims and procedures and provided their informed consent to allow data collection for research purposes.

### 2.3. Statistical Analysis

SPSS version 19.0 (IBM, Armonk, NY, USA) was used to perform statistical analysis. Quantitative data are presented as mean ± SD, frequency, and percentage. For continuous variables such as gestational age, age, and endometrial thickness, the Student’s *t*-test was used to highlight differences between groups, while for categorical variables such as the presence of a rich vascular pattern, the chi-square test was used.

The influence of each indicator was assessed using a binary logistic regression analysis. *p*-values < 0.05 were considered indicative of a statistically significant difference.

## 3. Results

From January 2016 to December 2022, 22 women had a diagnosis of CSP at the Cannizzaro University Hospital of Catania (Kore University of Enna—Italy). Among these, 19 patients were enrolled in the study because they met the selection criteria, while 3 patients were excluded as they had a primitive cervical pregnancy.

Women’s ages ranged from 26 to 43 years, with a median age of 34.74 ± 4.4 years. The mean gravidity was 4.3 ± 1.8 (range 2 to 9). Considering the number of cesarean sections before CPS, 6 patients (32%) had one cesarean section, 5 (26%) had two, 5 (26%) had three, 2 (11%) had four, and 1 patient (5%) had five previous cesarean sections. The mean gestational age was 8.0 ± 1.6 (range 6 to 12.3 weeks).

The ß-HCG level ranged from 7187 to 141,416 with a mean of 54,215 ± 47,297 IU/L, while the mean diameter of the gestational sac was 2.47 ± 1.01 cm (range 1 to 5 cm) and the mean endometrial thickness was 3.1 ± 1.39 mm (range 1.8 to 6 mm).

Table 1 summarizes the clinical characteristics of the sample, in relation to the treatment carried out. Among patients, 9 underwent treatment with MTX (47%), while 10 patients underwent treatment with UAE + D&C (53%). No significant differences were found between the 2 groups in terms of patients’ mean age, gestational age, gravidity, parity, previous cesarean sections, and the time interval between cesarean delivery and CSP.

Myometrial thickness was measured on average 3.2 ± 1.24 mm in the MTX group and 3.14 ± 1.59 mm in the UAE + D&C group, respectively. Patients treated with MTX had a mean diameter of the gestational sac of 2.1 ± 0.79 cm, those treated with UAE + D&C had a diameter of the gestational sac of 2.82 ± 1.10 cm. Fetal cardiac activity at the time of diagnosis was present in 7 (77%) patients in the MTX group and 7 (70%) patients in the UAE + D&C group.

In the MTX group, there were 3 (33%) cases of CSP type I and 6 (64%) cases of CSP type II, while in the UAE + D&C group, the cases of CSP type I were 5 (50%) and 5 (50%) of CSP type II.

The mean bHCG levels were 56,808 ± 56,251 for the MTX group and 51,299 ± 38,454 for the UAE + D&C group, respectively. Furthermore, 8 (88%) patients treated with MTX and 9 (90%) patients treated with UAE + D&C had a rich vascular pattern (RVP) in the scar. No statistically significant difference was found between the two groups regarding bHCG levels, mean endometrial thickness, presence of RVP, CRL, presence of FCA, and type of CSP, while the MTX group presented a significantly lower mean diameter of the gestational sac (*p* < 0.05) compared to the UAE + D&C group.

In the MTX-treated group, the mean dose of MTX administered was 1.6 ± 0.73 (range 1 to 3 doses).

Of the 9 patients in this group, 4 (44%) were successfully treated and did not require further treatments. In the remaining 5 (56%) patients of the group, there was permanence of FCA or growth of CRL or an unsatisfactory decline in bHCG levels after the first line treatment, for which they required further therapies. Of these, three patients following treatment with multiple doses of MTX underwent UAE + D&C, thanks to which they ultimately achieved a successful outcome.

One patient, after the first injection of 50 mg of MTX into the ovular cavity (day 1), required a further 25 mg of MTX (day 2) and subsequent operative hysteroscopy with the removal of the gestational sac (day 16) due to the persistence of an FCA.

Another patient, after the first injection of 50 mg of MTX into the ovular cavity (day 1), underwent an injection of a further 50 mg of KCL + MTX (day 9), and subsequent operative hysteroscopy for the removal of the gestational sac (day 19) following which a hemorrhage occurred as a complication, requiring first a blood transfusion and mechanical hemostasis with a Foley catheter in the uterus and then a total hysterectomy with bilateral salpingectomy, due to persistent uterine bleeding.

The main complications that occurred among women in this group were hemorrhage and pelvic pain, found in 2 patients (22%) of the 9 total. In the UAE group, however, the success was 100% given that all the patients required a single procedure, and only one patient experienced as a complication the presence of blood clots in the uterine cavity and the uterine isthmus and subjectively poor pelvic pain.

The mean length of hospital stay was 14.22 ± 6.38 days for the MTX group and 3.40 ± 1.65 for the UAE + D&C group, and this difference was statistically significant (*p* < 0.05).

The results obtained in the two groups are summarized in Table 2.

## 4. Discussion

The incidence of scar pregnancy has increased in recent times to 0.09% of all pregnancies, especially in high-income countries, due to the increased rate of cesarean sections and the low rate of vaginal deliveries after cesarean sections [15].

Despite different types of surgical and non-surgical approaches available to treat this life-threatening medical condition, there is no unanimous consensus on the best treatment to be offered to these patients.

In the present study, 88% (*N* = 16) of patients with CSP were successfully treated without any complications with two different techniques.

According to a recent comparative analysis of treatment options based on data obtained by the International Registry, a cesarean scar pregnancy can be managed successfully in the first trimester of pregnancy in almost all the patients (90%) with either suction evacuation, balloon treatment, or surgical excision [16]. The CSP International Registry is an international research project for data on CSP composed of an international network of centers (9 public hospitals, 21 academic institutions, and 2 private clinics) involved in the diagnosis and treatment of these patients [16]. Each participating center obtained authorization from a local ethical committee, and the examiners declared that they had a high level of experience and expertise in gynecologic ultrasound. The registry was created in 2018 to study the diagnosis and management of CSP pregnancies. The database contains retrospective data on patients affected by CSP, including data on anamnestic history, demographics, and previous pregnancies. ultrasound findings, management, and outcomes. Each center uploaded an ultrasound image showing the pregnancy, uterus, and endocervical canal in a longitudinal section and the Board of Registry reviewed them.

The effectiveness of all treatment options decreases with advanced gestational age; therefore, cesarean scar pregnancies should be managed as early as possible after confirmation of the diagnosis. Local medical treatment with potassium chloride or methotrexate is less efficient and has higher rates of complications than the other treatment options. Systemic methotrexate has a substantial risk of failing and a higher complication rate and should not be recommended as a first-line treatment [16]. However, the use of methotrexate injection, either systemic and/or local, can be an effective method, especially in patients with elevated β-HCG levels [17].

In our study, the success of treatment through a single procedure differed significantly between the two groups, highlighting the greater efficacy of treatment with UAE + D&C in inducing complete abortion after a single procedure (*p* < 0.05) and also considering the greater diameter of the gestational sac registered in the UAE + D&C group. Similarly, the difference in the number of patients who showed permanence of FCA, growth of CRL, or reduced decline in ß-HCG after the first treatment was significantly lower in the UAE + D&C group compared to the MTX one.

Among patients who reported complications, only 1 had severe complications that required demolitive surgery. This complication rate seems similar to other reports [16].

Currently, the main method of CSP diagnosis is the transvaginal US, either traditional two/three-dimensional Doppler US or contrast-enhanced US, which provides information about the gestational sac (size and shape), the thickness of the myometrium around the scar, the embryo or yolk sac, and the heartbeat. Recently, the procedure for evaluating the CSP during the first trimester of pregnancy was published. It includes 4 steps: 1—determination of location of the pregnancy: intrauterine pregnancy, low-implanted pregnancy, CSP, or miscarriage; 2—determination of type of CSP depending on whether the largest part of the GS is crossing the uterine cavity line; 3—determination of location of the placenta: in the niche, near the niche, or placenta previa; and 4—evaluation of the presence of signs of an abnormally adherent placenta [18]. Other diagnostic tools were recently submitted; for example, according to the relationship between the endometrial line and the superior-inferior (S-I) diameter of the ectopic sac, Calì et al. introduced a new sonographic sign, the crossover sign (COS) [19]. Cai et al. proposed a comprehensive clinical and sonographic classification scoring system to define the best treatment choice. For patients with a total score of ≥4 points, CSP is suggested, and early clinical treatment can be carried out, while patients with a score of less than 4 points can temporarily retain pregnancy and be closely followed up [20]. In our series, we used diagnostic criteria described by Timor-Tritsch, which were already published at the time we performed the study, moreover, these criteria are universally known as easy and simple principles in order to perform the diagnosis of cesarean scar pregnancy [12].

Dilatation and curettage in patients affected by cesarean scar pregnancy can be complicated by massive hemorrhage, so concomitant use of UAE was introduced in order to reduce the risk of subsequent treatments and to improve the rate of success.

In our series, patients treated with this technique obtained a full recovery (100%) without the need for further and more invasive procedures. Discomfort and pelvic pain caused by the ischemia after UAE was reported, but this complication can be prevented by positioning an epidural catheter to administer anesthetic drugs after the procedure. In our experience, this practice was progressively used, and nowadays it represents a standard procedure performed in association with the UAE.

In another retrospective study [21], patients diagnosed with CSP between January 2009 and December 2019, were treated with different treatments for each type of CSP with the objective to develop a management strategy for CSP. Local and systemic MTX administration success rates were 88.9% for type Ia and 66.7% for type Ib. Dilation and curettage, after uterine artery embolization and hysteroscopic resection were 100% successful.

A possible criticism of our study is the potential fertility damage in patients submitted to UAE.

A retrospective study compared three different treatment strategies: local or systemic methotrexate injection and surgery (MTX + Surg), uterine arterial embolization and surgery (UAE + Surg), or only surgery [22]. The incidence of intrauterine adhesions was highest in the UAE + Surg group (20%) when compared to the MTX + Surg group (0%), and the only surgery group (0%). In another study, authors evaluated reproductive outcomes in three groups of patients with CPS treated with expectant management, systemic MTX, and UAE combined with MTX and concluded that both therapeutic strategies have been effective in maintaining a normal reproductive potential [23]. Fertility preservation in women with CSP is a topic of pivotal relevance; therefore, future well-designed studies are required to understand the impact of different therapeutic approaches on patients’ fertility.

A limitation of the current study was its retrospective nature, determining a possible bias selection. Also, the absence of a standardized protocol for the administration of MTX in CSP could represent a bias.

The strategy did not include all the effective treatments reported in the literature, for example, the recent description of the intrauterine pressure balloon [24]. Another limitation is the small sample size, but these data can be available for further reviews and meta-analysis. Thus, a prospective, multicenter, randomized controlled trial is still needed to reach more definitive conclusions. Anyway, to our knowledge, this is the first study comparing the aforementioned treatments for the management of CSP, and this can be our strength.

## 5. Conclusions

The management of patients with CSP should be individualized according to differences in patient characteristics. However, timely detection and appropriate treatment are critical to achieving the most successful outcome. In our experience, the administration of MTX is not recommended as the primary treatment or pre-treatment. Dilatation and curettage after uterine artery embolization are better than methotrexate injection for the treatment of cesarean scar pregnancy in a single-institution series in terms of complete recovery and length of hospital stay.

We encourage the importance of the International Registry as a tool to improve the diagnosis, treatment, and outcome of CSP.

## Figures and Tables

**Table 1:** Clinical features of the study population.

	MTX Group (N = 9)	UAE + D&C Group (N = 10)	*p*-Value
**Age (years)**	35.5 ± 3.68	34 ± 5.10	0.23
**Gestational age (weeks)**	7.8 ± 1.67	8.18 ± 1.69	0.31
**Gravidity**	4.1 ± 1.54	4.5 ± 2.12	0.33
**Parity**	2.7 ± 1.58	2.3 ± 1.25	0.29
**Previous CD**	2.4 ± 1.42	2.2 ± 1.03	0.34
**Time interval (year)**	3.7 ± 1.73	5.22 ± 2.95	0.10
**Myometrial thickness (mm)**	3.2 ± 1.24	3.14 ± 1.59	0.48
**RVP**	8 (88%)	9 (90%)	0.25
**Mean sac diameter (cm)**	2.1 ± 0.79	2.82 ± 1.10	0.05
**CRL (mm)**	6.7 ± 7.21	12.43 ± 11.99	0.11
**FCA**	7 (77%)	7 (70%)	0.24
**CSP type 2**	6 (64%)	5 (50%)	0.44
**Level Bhcg (IU/L)**	56,808 ± 56,251	51,299 ± 38,454	0.41

Values are presented as mean or number (%). Abbreviations: UAE Uterine artery embolization, D&C dilatation, and curettage, CD cesarean delivery, RVP rich vascular pattern, FCA fetal cardiac activity, Bhcg beta human chorionic gonadropin. The *p*-value shows differences among groups (*p* < 0.05).

**Table 2:** Outcomes in the study population.

	MTX Group (N = 9)	UAE + D&C Group (N = 10)	*p*-Value
**Decline of Bhcg (%)**	71.4 ± 0.24	78.3 ± 0.13	0.27
**Successful cases**	4 (44%)	10 (100%)	0.01
**Nr. MTX doses**	1.6 ± 0.73	N.A.	-
**Permanence of FCA pr growth of CRL and Bhcg after 1st line therapy**	5 (55%)	0 (0%)	0.01
**Complications**	2 (22%)	1 (10%)	0.25
**Length of hospital stay**	14.22 ± 6.38	3.4 ± 1.65	0.0004

Values are presented as mean or number (%). Abbreviations: UAE Uterine artery embolization, D&C dilatation, and curettage, Bhcg beta-human chorionic gonadotropin, N.A. not applicable, FCA fetal cardiac activity, CRL crown-rump length. The *p*-value shows differences among groups (*p* < 0.05).

## Data Availability

The data that support the findings of this study are available from the corresponding author upon reasonable request.
